# From Simulation to Therapy: Light Guidance Through Zirconium Dental Implants for Bone-Targeted Photodynamic Inactivation

**DOI:** 10.3390/bioengineering13080880

**Published:** 2026-07-30

**Authors:** Kolja Lehmann, Gabor Kadler, Heinrich Walt, Barbara Solenthaler, Harald Essig, Michael Guthe

**Affiliations:** 1 Department of Cranio-Maxillo-Facial and Oral Surgery, University Hospital Zurich, University of Zurich, 8091 Zurich, Switzerland; 2 Department of Computer Science, ETH Zurich, 8092 Zurich, Switzerland; solenthaler@inf.ethz.ch; 3 Faculty of Mathematics, Physics and Computer Science, University of Bayreuth, 95447 Bayreuth, Germany

**Keywords:** periimplantitis, zirconium dental implants, photodynamic inactivation (PDI), Monte Carlo Simulation of Radiation Transport (MCRT), interspecies difference

## Abstract

Bacterial biofilm formations on dental implant–bone borders risk the development of periimplantitis (PI). Treatment, ranging from conservative to invasive options, is effective but associated with significant patient burden. In vitro Photodynamic Inactivation (PDI) waveguided by zirconium dioxide ceramic (ZrO_2_) dental implants shows promising bactericidal effects and is envisioned to function as an additive treatment reducing patient burden while maintaining therapy outcomes. To facilitate clinical application, this study mathematically quantified the expected waveguiding abilities of ZrO_2_ implants in a computed tomography (CT)-based Monte Carlo Simulation of Radiation Transport (MCRT). The latter implemented previously photographically determined optical properties from human and porcine jawbone tissues. ZrO_2_ implants displayed effective light propagation beyond the implant–bone border, with power-per-area values above a minimum baseline for effective PDI of 10 mW/cm^2^, thus delivering enough light power to reach bactericidal levels in a standard PDI regimen. Modifying the implant design and light-source placement influenced the topographic distribution of the light power around the implant, eventually allowing to target case-specific changes in biofilm distribution. No major interspecies difference was observed, which fosters the transferability of potential mammal testing. All findings therefore support the envisioned application of waveguiding ZrO_2_ dental implants in PDI to target oral health issues.

## 1. Introduction

Development in dental implantology has led to an increase in the number of inserted dental implants, with an estimated prevalence of 23% in 2026 [1]. Although surgical implantation techniques have strongly improved implant survival rates, implant failure due to periimplantitis (PI) is still common [2,3]. PI is associated with intraoral bacterial biofilm formations on the implants and infection of the surrounding tissue [4,5]. Biofilm is extensively found on the supramucosal implant parts, but research suggests that untreated PI might progress along the implant shaft into the intraosseous part, colonizing an increased proportion of the implant–bone border [6,7,8,9]. This progression risks the development of potentially grave osteomyelitis of the jaw [10,11]. With intraosseous biofilm being a risk factor for aggravated infections, treatment of early PI stages ranges from antimicrobial rinses to invasive surgical implant removal with the need for subsequent reconstructive surgeries [12,13]. These extensive treatment regimens risk surgical complications and long healing periods [14,15]. To reduce this therapy burden on patients, research has focused on the innovative usage of Photodynamic Inactivation (PDI) to address bacterial biofilm and prevent the exacerbation of early-stage PI [16,17]. With successful implementation in other medical fields, such as dermatology and interventional radiology, PDI is considered a safe antibacterial therapy, with recent studies demonstrating reliable bactericidal efficacy [18,19,20,21,22]. Common PDI protocols implement a 670 nm red-light laser to excite a Methylene Blue (MB) photosensitizer at its excitation maximum in the range of 670 nm, which, in return, induces the formation of cytotoxic reactive oxygen species (ROS) [23,24,25,26]. ROS damage bacterial cell walls, leading to biofilm destruction via bacterial cell death [27,28,29]. Prevailing dental implant materials titanium alloys (Ti) and zirconium dioxide ceramics (ZrO_2_) show no significant difference in susceptibility to biofilm formation, but in vitro ZrO_2_ material cultivated with bacterial biofilm has displayed superior antimicrobial properties during MB-based PDI compared to Ti controls [30,31]. In our preceding work, we found ZrO_2_ to facilitate significant bactericidal effects during MB-based PDI performed with low energy densities of 15 J/cm^2^ [31]. The in vitro PDI effectiveness of ZrO_2_ is attributed to its waveguiding properties allowing light to reach bacteria hidden from direct illumination through transmission and potentially targeting intraosseous biofilm formations around the implant–bone border [8,9,32,33]. Addressing this major cause for severe disease progression is believed to significantly improve the treatment outcome of PI.

As previous publications have highlighted the in vitro feasibility of ZrO_2_ dental implants as waveguides in PDI, the future aim is clinical testing. To bridge the gap between in vitro results and potential applications in animal and human test subjects, this study models, for the first time, the expected ZrO_2_ waveguiding abilities for porcine and human tissue data in a novel in silico PDI computer simulation. The aim was to quantify the expected light propagation of ZrO_2_ dental implants in the mandible region for the mentioned 670 nm wavelength, envisioning a MB-based PDI regimen and allowing for the investigation of possible interspecies differences. To model the light pathway in this complex region, we developed an optimized mathematical Monte Carlo Simulation of Radiation Transport (MCRT). The latter implement repeated random sampling to predict the pathways of photons through different tissue types with respect to their optical properties [34,35,36,37]. A hardware-accelerated path tracer was incorporated to optimize the simulation time [38]. The MCRT was combined with a CT dataset of a human viscerocranium to render an additional 3D model of the predicted light power. While similar approaches have recently been tested in the PDI treatment planning of glioblastoma and non-melanoma skin cancer patients, this study’s innovative combination of the MCRT and a CT dataset represents a unique approach in maxillo-facial research [39,40]. To allow for preclinical optimization, light-source position and implant design could be modified in the adaptable MCRT. Prior to the simulation, we determined the optical properties of human and pig mandibular bone pieces, human gingiva and the ZrO_2_ implant material in photographic experiments. Incorporating these findings into the MCRT guaranteed accurate representation of in vivo oral circumstances for both species.

## 2. Materials and Methods

In this study, we have modeled how PDI is waveguided by ZrO_2_ dental implants in the mandible region. The MCRT incorporated the optical properties of the implant material and jaw tissue from preceding photographic experiments. The implant material was analyzed from a ZrO_2_ disc (diameter: 10 mm; height: 2 mm), while the investigated tissue layers comprised an ex vivo human mandibular bone piece extracted from a young female patient undergoing bimaxillary osteotomy (length: 18.9 mm; width: 4.9 mm; height: 2.1 mm) and a pig mandible bone piece (length: 19.2 mm; width: 9.3 mm; height: 3.7 mm). The postmortem pig mandible was received immediately after slaughter from the veterinary service of Zurich. Overall short durations of under 30 min between extraction and experiment reduced the effect of normal ex vivo tissue decay, enabling a reliable model of in vivo circumstances [41,42]. Further, the human in vivo frontal mandibular gingiva tissue of a volunteering individual was incorporated into this study. The Cantonal Ethics Commission (KEK) in Zurich, Switzerland waived any needed approval for the investigated tissue (KEK BASEC-ID: Req-2026-00052).

### 2.1. Photographic Experiments

The MCRT simulation of light propagation in the lower jaw region relied on multiple optical properties of the involved ZrO_2_ implant material, mandible bone and overlying gingiva tissue. Therefore, the photographic experiments determined the absorption coefficient (µ_a_), the scattering coefficient (µ_s_) and the anisotropy of the phase function (g) for the investigated specimen. With respect to the governing physical laws, those constants were used to calculate the reduced scattering coefficient (µ_s_′), the extinction coefficient (ε) and the mean free path (λ) for the photons passing through the different tissue layers. Reduced scattering is defined as the mathematical combination of µ and the anisotropy of phase function g, representing how light scatters through non-transparent media, such as the investigated tissues [43,44]. Summing up the µ_a_ and µ_s_ results in the extinction coefficient ε quantified the total attenuation of light passing through the tissue type. The mean free path, λ, is the reciprocal fraction of ε and determines how far a photon will propagate until it is either scattered or absorbed within a specific medium.

To visually determine those optical properties for the envisioned MB-based PDI protocol with an excitation maximum in the range of 670 nm, the measuring setup comprised a 670 nm red-light laser with a transparent waveguide tip operating at 95 mW (Orcos Medical AG, Küsnacht, Switzerland) [23,25,26]. Further, a pinhole aperture, the implant disc or tissue specimen and a commercially available Sony camera (DSC-RX100M3) (Sony Corporation, Tokyo, Japan) were used. The influence of distorting background light was reduced by placing the measurement setup in complete darkness. The laser and the investigated implant material or tissue were positioned in a line 20 cm apart from each other, with the hole aperture centered in between, to focus the laser light onto the specimen while reducing the influence of scattered light. The camera was set into uncompressed RAW imaging mode (ISO-100, Aperture F/1.8), allowing for conversion of the light intensity of the picture into SI units such as power per area (W/cm^2^). For each measurement involving the ZrO_2_ disc or mandible, the camera was positioned at 45° and 135°, in respect to the radiation beam, to detect the laser light on the front as well as on the backside of the investigated object while preventing direct reflection of the laser. After image rectification, the scattering profiles on both sides were used to calculate µ_a_, µ_s_ and g using the MCRT simulation and a non-linear downhill simplex search [45]. The pig mandible allowed for separate optical measuring of the cortical and trabecular mandible parts, while the human bone counterpart was investigated in toto. As the MCRT focused on the implant–bone border light propagation, the optical influence of the peripheral soft tissue in the jawbone region on the PDI simulation was reasoned to be marginal. The optical properties of the human gingiva were therefore used as an adequate surrogate in the MCRT. With the vital human gingiva being investigated intraorally, the optical properties had to be determined via the back-scattering profile at the camera position of 45°. As the pig mandible did not allow for any investigation of a gingiva counterpart, the human gingiva was additionally implemented in the pig simulation.

### 2.2. CT-Based Monte Carlo Simulation of Radiation Transport (MCRT)

The CT-based MCRT modeled the light propagation through the waveguiding implant and adjacent tissue based on the determined optical properties. As a result, the expected photon number at the implant–bone border and thus the potential intraosseous biofilm could be determined for future PDI application. A previously performed human-head CT scan functioned as an anatomical reference for the subsequent 3D visualization of the human and porcine simulation, with the implemented optical properties differing between the two species based on the photographic experiments. The simulation involved a conventional solid ZrO_2_ implant (length: 8.5 mm; ø: 4.4 mm) and another innovative implant (length: 8.5 mm; ø: 4.4 mm) with an additional central channel of a 1.3 mm diameter exclusively designed for this study (Zircon Medical AG, Altendorf, Switzerland). Implants were computationally inserted 9 mm into the lower-right mandibula at dental position 44 of the CT model. The red-light laser (670 nm, 95 mW) from the photographic experiments was used to simulate the light source for a standard MB-based PDI protocol. Combined with the experimentally defined optical properties (µ_a_, µ_s_, g), the simulation calculated the paths of one billion photons passing from the tip of the laser waveguide through the implant and tissue layers [38]. An angular emission profile of the waveguide was estimated from the photographic experiments and verified with theoretical distributions from optical fibers. The resulting 3D model visualized the expected power per area (mW/cm^2^). This allowed for the investigation of the following parameter combinations (PCs) in both species: PC 1 (conventional solid implant with radiation placed on top of the implant), PC 2 (innovative implant with radiation placed on top of the implant), and PC 3 (innovative implant with radiation inserted 8 mm into the central implant channel). Subsequent conclusions on the effects of implant design and radiation positioning as well as interspecies differences could be drawn from the gathered data.

### 2.3. Mathematical Data Analysis

The MCRT data was visualized in a 3D color-coded model showing the topographic distribution of light power per area (mW/cm^2^) for the 670 nm wavelength. A mean radius (*R*) was defined to determine the average distance from the light source to all surface points of the volume with a certain power-per-area threshold between 0.1 and 90 mW/cm^2^. The obtained values of *R* in respect to the underlying threshold were used to calculate an approximation curve. This enabled an objective quantification of light propagation depending on the parameter combination and species model.

## 3. Results

### 3.1. Photographic Experiments

**Figure 1:**
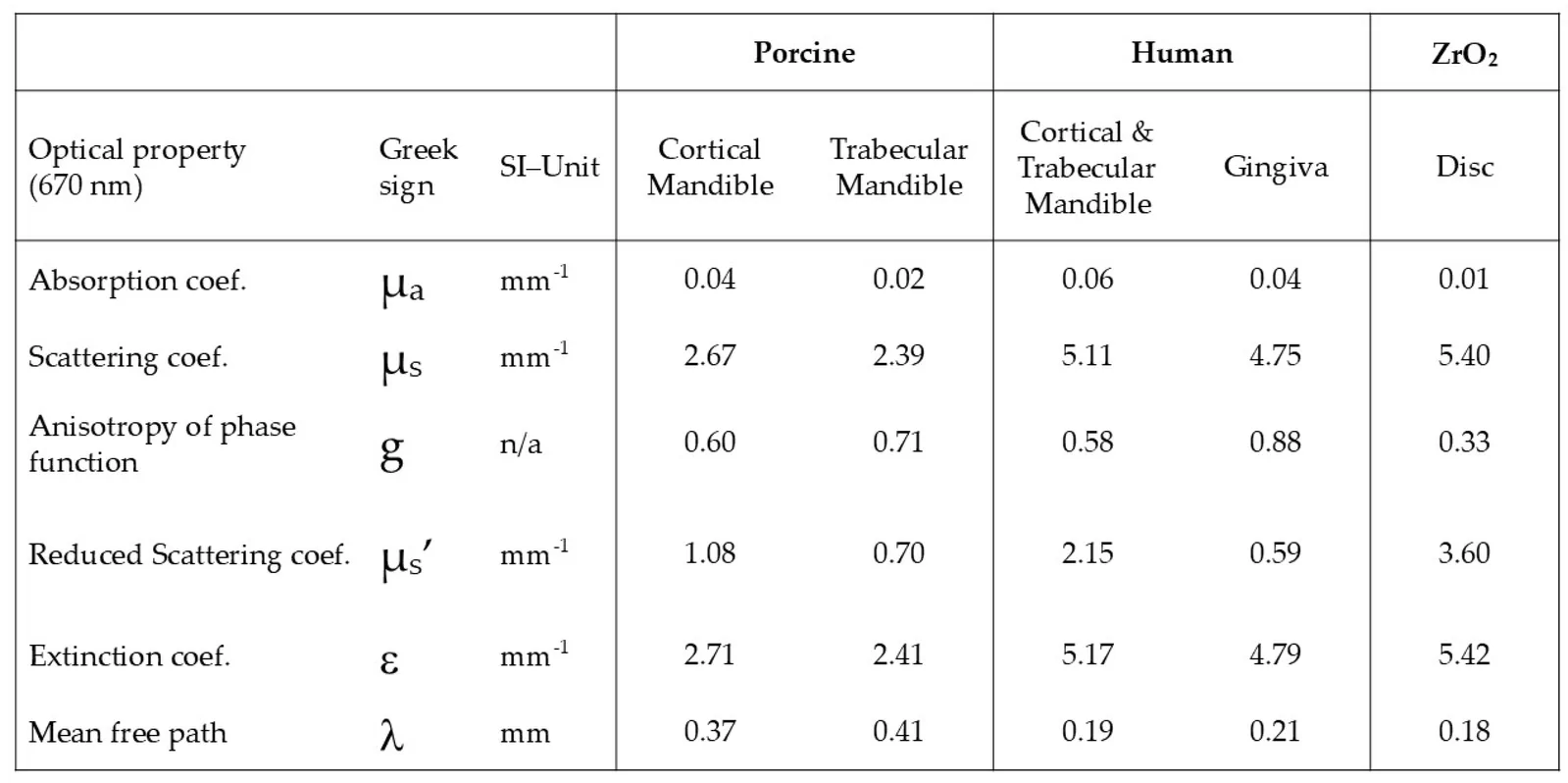
Optical properties determined for a red-light laser wavelength of 670 nm.

The present study developed a novel CT-based MCRT algorithm to model the light propagation of potential PDI protocols waveguided by ZrO_2_ dental implants in the mandible region. It therefore relied on experimental optical properties of the involved tissue types, such as the mandible bone and gingiva. The parameters of interest (µ_a_, µ_s_, g, µ_s_′, ε, λ) were successfully determined for the red-light laser wavelength of 670 nm (Figure 1). The highest absorption was observed in the human mandible, with 0.06 mm^−1^, while the waveguiding ZrO_2_ material demonstrated the lowest, with 0.01 mm^−1^. The absorption coefficients in the pig cortical bone and human gingiva were both 0.04 mm^−1^. The strongest scattering was observed in the ZrO_2_ material, with 5.40 mm^−1^ and the weakest in the pig trabecular mandible, with 2.39 mm^−1^. The scattering coefficient in the human mandible was 5.11 mm^−1^, and in the human gingiva, 4.75 mm^−1^. The highest anisotropy of a phase function was observed in the human gingiva, with 0.88, followed by the porcine trabecular bone, with 0.71. The porcine cortical and human bones were similar, with 0.60 and 0.58, respectively. The ZrO_2_ material displayed the lowest g, with 0.33. Mathematically combining µ and g resulted in the reduced scattering coefficient, µ_s_′, with the highest value obtained in the ZrO_2_ material, at 3.60 mm^−1^, followed by the human mandibula specimen in toto at 2.15 mm^−1^. The lowest µ_s_′ was detected in the human gingiva, with 0.59. Summing up µ_a_ and µ_s_ led to the extinction coefficient, ε, with the highest value seen in the ZrO_2_ material, with 5.42 mm^−1^, and the lowest in the pig trabecular bone at 2.41 mm^−1^. The human bone and gingiva both showed ε values of around 5 mm^−1^. mostly due to the corresponding high scattering coefficients, µ_s_. Ultimately, the mean free path, λ, was calculated to determine the interaction-free path a photon propagates through the investigated tissue. The longest λ was observed in the pig trabecular bone, with 0.41 mm, and the lowest in the ZrO_2_ material, with 0.18. The human gingiva and mandible also showed small λ values, with 0.21 mm and 0.19 mm, respectively.

### 3.2. MCRT Simulation

**Figure 2:**
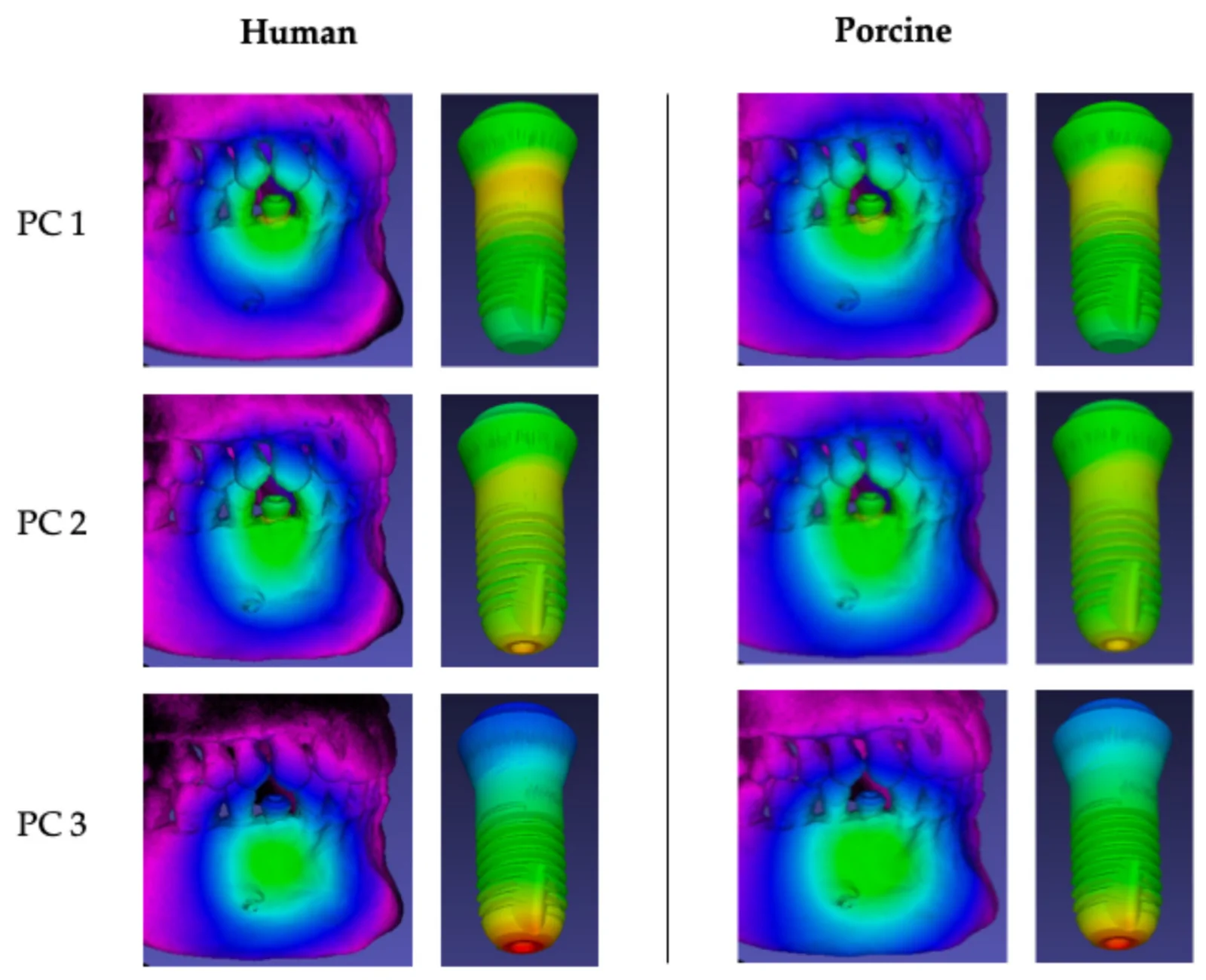
MCRT power-per-area topographic model of bone and implant for each parameter combination in human and porcine simulations with 95 mW (red: 1000 mW/cm^2^; yellow: 100; green: 10; turquoise: 1; blue: 0.1; violet: 0.01), visualized in open-source MeshLab 2025.

The MCRT modeled the light propagation from the waveguide tip through the ZrO_2_ implant and adjacent tissue layers. The resulting six topographic models of power per area (mW/cm^2^) visualized the difference between the parameter combinations in the human and porcine simulations (Figure 2). The model was color-coded, with the laser light source being calculated to have a starting power per area of 1000 mW/cm^2^. Within both species, all three PCs displayed notable light propagation beyond the implant diameter, and the effect of changing PCs led to similar topographic results. PC 1 in the human and porcine model depicted a spherical light propagation, with 10 mW/cm^2^, reaching both adjacent teeth 43 and 45. The corresponding solid implant topographically showed a power per area ranging between 10 and 100 mW/cm^2^. This converts to a factor-10–100 reduction in power per area through the absorption and scattering of the implant. PC 2 facilitated oval bone propagation with increased depth penetration compared with PC 1. In line with the stronger depth penetration, the channeled implant displayed a power per area of 100 mW/cm^2^ at its inserted tip, as light radiating in a straight path through the channel only minimally interacted with the implant. In PC 3, the depth penetration was increased more by inserting the light source 8 mm deep into the implant channel. As a result, the spherical propagation was transferred vertically and confined to the mandible in the human simulation while the porcine model even propagated light of 1 mW/cm^2^ to the roots of 43 and 45. Both implants led to strong power-per-area gradients, with the highest value, of ≥1000 mW/cm^2^, observed at the tip resulting from the interaction-free light propagation within the channel.

**Figure 3:**
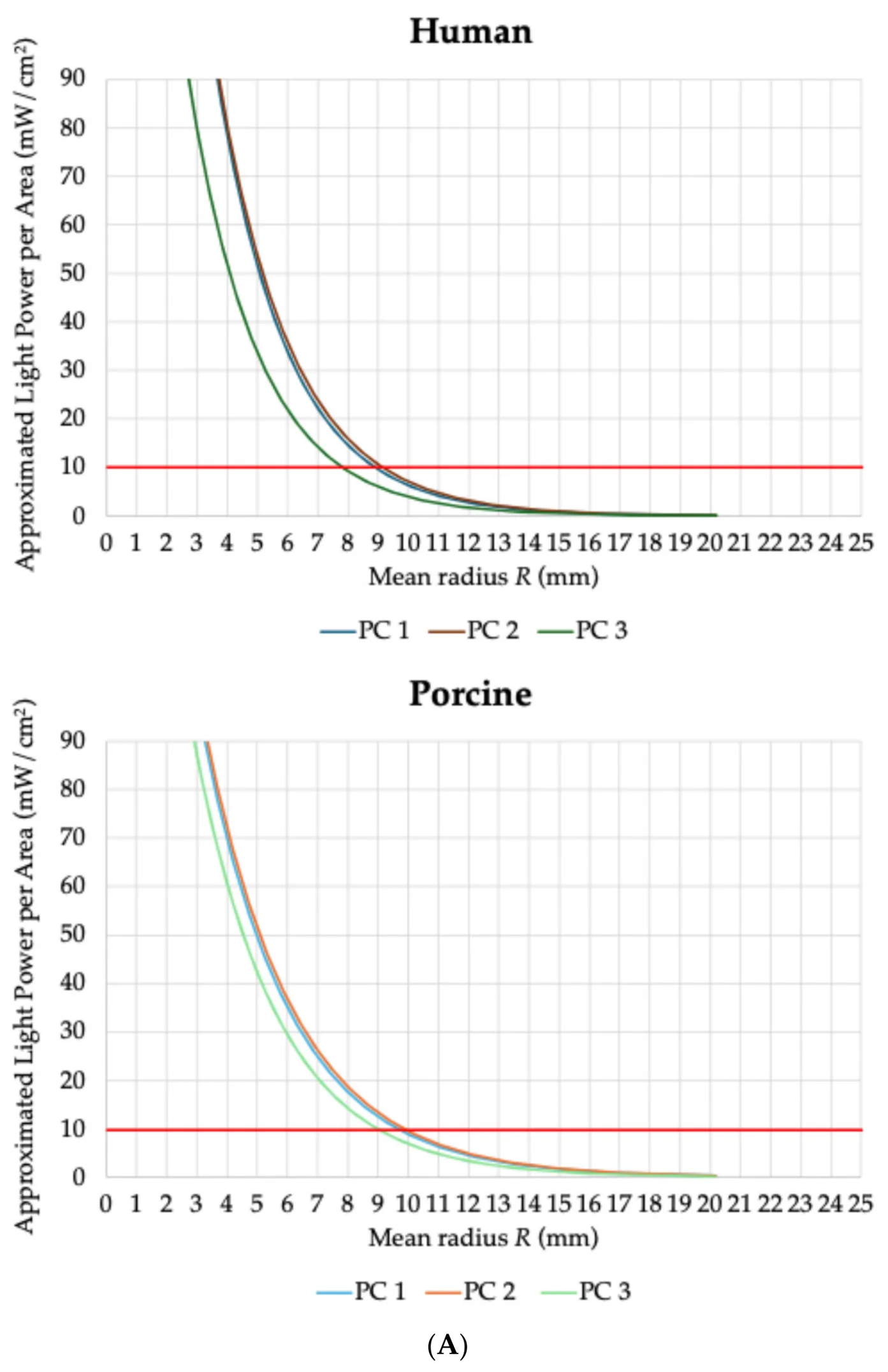

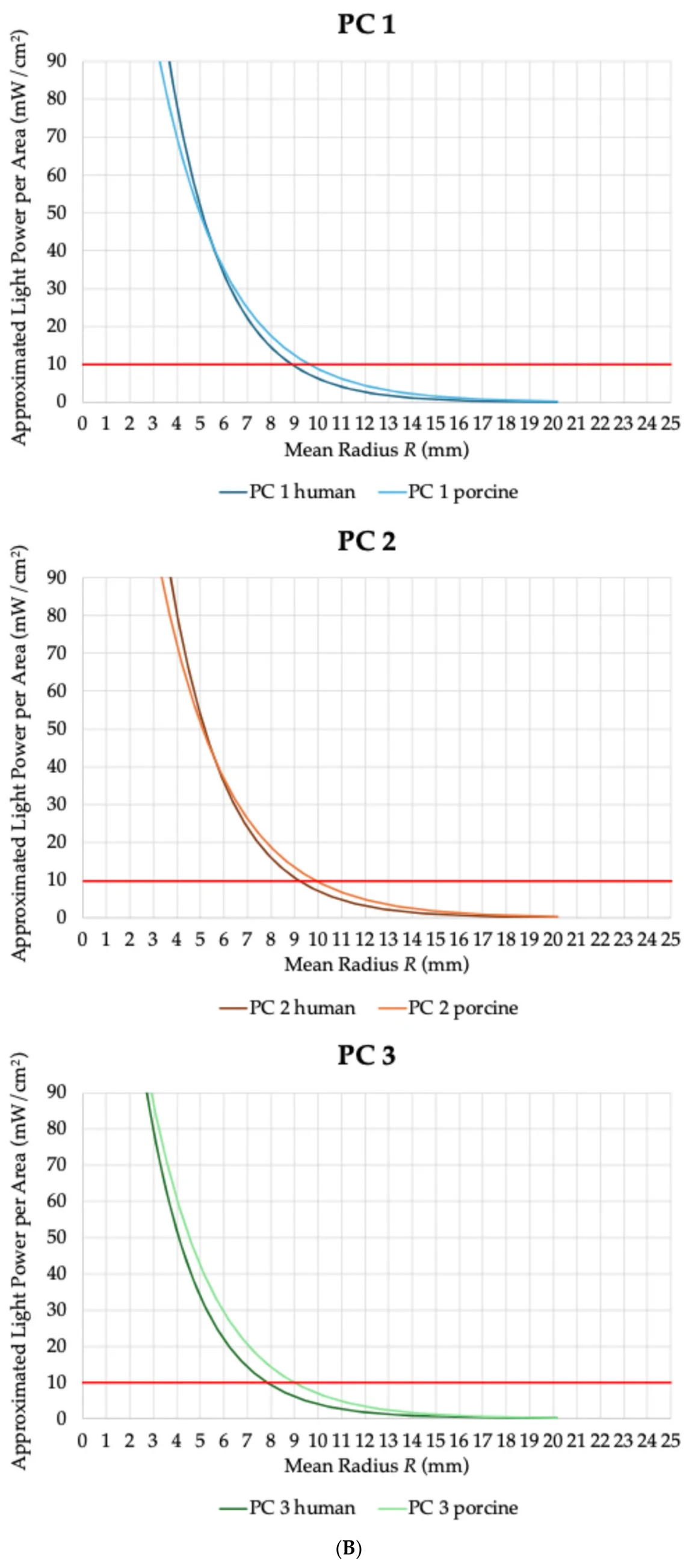
(**A**). Approximation curves of light propagation by species for 95 mW, with a red line indicating the 10 mW/cm^2^ threshold. (**B**). Approximation curves of light propagation by parameter combination for 95 mW, with a red line indicating the 10 mW/cm^2^ threshold.

The MCRT visualization confirmed the effective light propagation through waveguiding implants and adjacent bone tissue. It therefore proofed the envisioned feasibility of waveguided PDI implementation in targeting bacterial biofilm at the implant–bone border. The simulation allowed for a mathematical approximation of the light propagation in respect to the parameter combinations and possible interspecies difference. The mean radius, *R* (mm), was calculated for all investigated power-per-area thresholds (Supplementary Table S1). The obtained values for *R* were used to determine the approximation curves derived from the six MCRT models (Figure 3A,B). According to recent in vitro findings on MB-based PDI, a minimum power-per-area value of ≥10 mW/cm^2^ could reduce the potential PDI duration to under 25 min while guaranteeing enough excitation energy for the MB photosensitizer to maintain bactericidal levels [31,46]. This value was therefore defined as a target threshold in the subsequent approximation curves, indicated by a red line. All six topographic models displayed favorable power-per-area values ≥ 10 mW/cm^2^ on most of the implant–bone border. The curves depicted an inverse exponential correlation between the mean radius, *R*, and the corresponding power per area from the laser light source (Figure 3A). The slope of the power-per-area value decreased with the increasing mean radius from the light source. For both species, the relationships between the three PC curves were similar. The approximation curves of PC 1 and 2 were only marginally different, with PC 2 depicting slightly larger *R* values for a specific power per area. PC 3 showed the least light propagation, with the smallest *R* for a defined power per area. The mean radius for the 10 mW/cm^2^ threshold was above 7.5 mm in all three human simulations and above 9 mm in the porcine counterparts. Based on these findings, the power-per-area approximation around the implant outer surfaces, with radii of 2.2 and 1.55 mm, respectively, were reasoned to be notably higher than the 10 mW/cm^2^ threshold. Further, the interspecies difference was smaller than expected from the optical properties (Figure 3B). PC 1 and 2 of both species demonstrated nearly identical approximation curves. Nonetheless, the curves marginally diverged above and below a threshold of 40 mW/cm^2^. PC 3 displayed the largest difference between the human and pig models, with mean radii for the threshold of 10 mW/cm^2^ of 7.81 mm and 8.99 mm, respectively.

## 4. Discussion

Periimplantitis is a frequent complication in dental implantology, with therapy approaches ranging from antibiotics to invasive surgical removal of the implant [2,3]. To reduce PI progression and therapy-associated patient burden, research has focused on the innovative usage of PDI to target the underlying bacterial biofilm [47,48]. While common titanium dental implants are non-transparent, innovative ZrO_2_ alternatives have shown light-scattering properties believed to be beneficial for PDI [31,49]. Recent in vitro findings suggest that ZrO_2_ dental implants could work as optical waveguides in PDI to potentially augment its effect along deeper layers of infected tissue [31,49]. To optimally plan a subsequent in vivo experiment, this study aimed at mathematically modeling the expected ZrO_2_ waveguiding effect in PDI with a Monte Carlo Simulation of Radiation Transport. MCRTs are respected simulations in multiple clinical fields of research but represent a new approach in the field of maxillo-facial surgery [39,40]. This unique MCRT quantified the light propagation through ZrO_2_ implants and adjacent bone tissue layers to determine the feasibility of a potential clinical application. It focused on the effects of light-source positioning as well as implant design and compared the PDI waveguiding effect between human and pig models. To enable an accurate simulation, the MCRT incorporated photographic experiments that determined the optical properties of the involved tissue layers in the human and pig specimens.

The photographic experiments obtained the optical properties of human and pig bone tissue as well as intraoral human gingiva for a laser wavelength of 670 nm. While the optical property, µ_a_, of the pig specimen was found to be in line with preexisting findings (0.001–0.1 mm^−1^), the literature suggests that the corresponding µ_s_ coefficient was larger than determined in our study [50,51]. For the human bone counterpart, µ_a_ exceeded the range from existing studies (0.005–0.3 mm^−1^), but the related reduced scattering coefficient, µ_s_′, only slightly exceeded the prevailing scientific value range [52,53,54]. The human gingiva, on the other hand, showed smaller µ_a_ and µ_s_′ values than have been previously found for mucous tissue [55]. Concerning the ZrO_2_ dental material, the determined optical properties fall within the existing range of light properties [56]. The overall observed discrepancy of optical properties with the prevailing literature is attributed to different experiment setups with varying light-source types and wavelengths, especially since this study focused on one wavelength of 670 nm. Further, specimen size, histological composition and tissue vitality linked to the biomechanical and metabolic processes might strongly change between investigated specimens and thus heavily influence the results of optical experiments [57]. Thus, comparison of optical properties to the existing literature is believed to be limited.

The experimentally determined optical properties were implemented in the subsequent MCRT algorithm. All six simulations indicated a strong waveguiding ability of ZrO_2_ dental implants, with power-per-area values ≥ 10 mW/cm^2^ observed up to 7 mm beyond the implant–bone border. Bacterial biofilm formations extensively cultivate implant surfaces in clinical periimplantitis [4,5,7]. As ZrO_2_ implants guarantee strong light propagation beyond this point, their waveguiding ability promotes the envisioned bactericidal effect. The latter also relies on the overall accumulated light energy to produce enough bactericidal ROS [23,24,27]. The required corresponding energy density varies depending on the targeted pathology as well as the applied PDI regimen, including light source and photosensitizer [27]. Thus, values largely differ in the scientific literature, ranging between 6 and 25 J/cm^2^ [58,59,60]. Focusing on enoral pathogens, recent studies have even shown effective PDI with energy densities at 15 J/cm^2^ [31,61]. Translated to the calculated power per area exceeding 10 mW/cm^2^ in the performed MCRT, the corresponding PDI time would account for less than 25 min, therefore representing a time-effective therapy option. Additionally, the implemented laser allows for a maximum initial light power of up to 350 mW while still working in a non-hazardous energy range (Orcos Medical AG, Küsnacht, Switzerland). Increasing the initial light power from the investigated 95 mW to the maximum power of 350 mW could further reduce the mentioned time needed for effective PDI.

The underlying three parameter combinations of the obtained MCRT show varying light propagation within both species. While PC 1 and 2 have nearly equal approximation curves, PC 3 displays a discretely left-shifted curve in comparison. Further, an apparent difference in light propagation topography in the CT model was found (Figure 2 and Figure 3). Identical in light-source placement on top of the implant head, the first two parameter combinations differ in implant design. PC 1 uses the solid implant and PC 2 the channeled one. The oval shape with vertical dominance in PC 2 is attributed to the direct light beam through the channel. The central hole permits a fraction of photons to cover a relatively long distance without energy loss through optical interaction. This influences the overall light propagation form and potentially increases the expected mean radius, *R*. In contrast, the solid implant in PC 1 provides more material volume for optical interaction, leading to increased absorption and scattering [62]. Energy loss through absorption likely reduces the mean radius, while scattering visibly maintains the observed power per area on the implant neck (Figure 2). The latter presumably causes the spherical light propagation of PC 1 in comparison with PC 2. Despite the topographic power-per-area differences, PC 1 and 2 have similar approximation curves. This indicates that the underlying change in implant design does not significantly influence the overall light propagation.

Advancing the light source 8 mm into the channel resulted in parameter combination 3. The corresponding topographic models exhibited a spherical light propagation centered in the inferior bone layers, with higher power-per-area values at the implant tip. This would allow the targeting of progressed PI stages where bacterial invasion has spread to deeper bone layers [6,7]. The overall implant surface showed a heterogenous power-per-area topography, with strongly decreasing values away from the tip. As biofilm can be dispersed around the whole implant–bone border, topographic differences in achieved light power might limit effective PDI and correlating therapy outcomes on superior implant and bone layers [6,8]. Further, the resulting leftward-shifted approximation curve of PC 3 suggests a reduced mean radius, *R*, in comparison with PC 2. Both the gradient and overall reduced approximation curve could be linked to the light-source positioning. The positioning in PC 1 and 2 allowed for a fraction of light to enter the tissue around the implant first before being scattered into the implant. As the whole light source was submerged in the implant for PC 3, light first interacted with the implant core before reaching its surface. With ZrO_2_ having a higher extinction coefficient and smaller mean free path than the surrounding bone tissue, more light energy is lost when photons travel from the implant inner core to the outer border than propagating in the opposite direction. The gradient on the topographic model could be linked to this optical interaction. It is therefore noted that the photon path and sequence of optical interaction influence the resulting calculated power-per-area value [62]. Additionally, positioning the light source within the channel reduced the mentioned effect of the direct light beam on the mean radius, *R*, which shifted the overall approximation curve to the left.

Based on those investigated parameter combinations, the interspecies difference was analyzed. The MCRT did not find an overall notable discrepancy between the two species, although the determined optical properties differed between the human and porcine bone specimens (Figure 3B). The approximation curves of PC 1 and 2 were nearly identical for the human and porcine specimens. Only PC 3 showed a slight difference, of around 1 mm, in the mean radius at 10 mW/cm^2^. As previously stated, for PC 3, inserting the light source into the implant channel limited the possible sequence of light propagation and therefore reduced the number of arbitrary photon pathways compared with PC 1 and 2. Thus, the slight interspecies difference is believed to have been masked in the first two parameter combinations while only becoming apparent in PC 3.

This study also demonstrated minor limitations. The optical properties were obtained from one specimen of each species only. It sufficed to model the waveguiding ability of ZrO_2_, but the obtained results might not be directly transferable to a general population. Increasing the sample size and averaging the determined optical properties in future experiments can counteract this limitation. Additionally, this model focused on the light propagation from the implant to the adjacent bone tissue. The effect of peripheral soft tissue such as fat, blood vessels, connecting tissue, muscles and skin was therefore assumed to be minimal, and the human gingiva was used as a surrogate. To counteract a potential data distortion and increase the simulation accuracy, additional optical investigations of other intraoral tissue components, such as saliva and mucus, as well as pathological inflamed tissue and potential biofilm formations, could be included [63,64,65]. Further, the MCRT light propagation model was planned while envisioning common PDI regimens involving a Methylene Blue photosensitizer and therefore focused on its excitation wavelength of 670 nm operating at 95 mW [23,27]. Investigating multiple light sources operating at different wavelengths and powers might enhance the clinical applicability to other PDI treatment protocols with different photosensitizers [66]. This study also limited its simulation to three parameter combinations. This allowed us to test the most anatomically reasonable PDI setups, but expanding the parameter combinations could reveal new insights as well as unmask subtle differences not seen in the current simulations.

## 5. Conclusions

In this study, we investigated the waveguiding ability of ZrO_2_ dental implants for potential PDI treatment of periimplantitis biofilm in the mandible. The aim was to determine its feasibility and facilitate the transition from in vitro testing to clinical trials. Representing an unprecedented approach in the maxillo-facial specialty, we combined photographic experiments that obtained optical properties from human and porcine mandible specimens with our own optimized mathematical CT-based MCRT simulations. The latter quantified the light propagation through ZrO_2_ dental implants to adjacent bone tissue to evaluate the expected bactericidal effect at the implant–bone border. The ZrO_2_ implants displayed effective light propagation at the implant–bone border of more than a 7.5 mm radial beyond the light source in both species, indicating the envisioned feasibility. With reached power-per-area values of notably higher than 10 mW/cm^2^, the standard clinical PDI treatment length could be reduced to less than 25 min. By adapting the implant design and light-source placement, topographic differences in light power per area were achieved. This would allow to target variations in biofilm dispersion.As a result, waveguided PDI could be suitable to address patient-specific disease differences in line with the goal of precision medicine advancements. The MCRT displayed a narrow interspecies difference between the human and pig models, indicating possible similarities and the transferability of potential mammal testing. All presented findings support the feasibility of ZrO_2_ dental implant waveguiding in PDI and allow optimized planning of potential in vivo experiments, thus fostering its envisioned position as an additive treatment in fighting oral health issues.

## Data Availability

The underlying data for the findings presented in this study are available in the Supplementary Materials.

## Supplementary Materials

The following supporting information can be downloaded at: https://www.mdpi.com/article/10.3390/bioengineering13080880/s1, Table S1: Underlying Data from MCRT for Approximation Curves; Table S2: Exponential fit Equations for Approximation curves determined from Table S1.

## Abbreviations

The following abbreviations are used in this manuscript:

CT	Computed tomography
MB	Methylene Blue
MCRT	Monte Carlo Simulation of Radiation Transport
PC	Parameter combination
PDI	Photodynamic Inactivation
PI	Periimplantitis
ROS	Reactive oxygen species
Ti	Titanium alloy
ZrO_2_	Zirconium dioxide ceramics

## References

[1] Elani H.W., Starr J.R., Da Silva J.D., Gallucci G.O. (2018). Trends in Dental Implant Use in the U.S., 1999–2016, and Projections to 2026. J. Dent. Res..

[2] Niedermaier R., Stelzle F., Riemann M., Bolz W., Schuh P., Wachtel H. (2017). Implant-Supported Immediately Loaded Fixed Full-Arch Dentures: Evaluation of Implant Survival Rates in a Case Cohort of up to 7 Years. Clin. Implant Dent. Relat. Res..

[3] Renvert S., Roos-Jansåker A.-M., Claffey N. (2008). Non-Surgical Treatment of Peri-Implant Mucositis and Peri-Implantitis: A Literature Review. J. Clin. Periodontol..

[4] Berglundh T., Armitage G., Araujo M.G., Avila-Ortiz G., Blanco J., Camargo P.M., Chen S., Cochran D., Derks J., Figuero E. (2018). Peri-Implant Diseases and Conditions: Consensus Report of Workgroup 4 of the 2017 World Workshop on the Classification of Periodontal and Peri-Implant Diseases and Conditions. J. Clin. Periodontol..

[5] Obreja K., Begic A., Ramanauskaite A., Schwarz F. (2020). PeriimplantitisDefinition, Symptome und leitliniengerechte Empfehlungen. Der Freie Zahnarzt.

[6] Dieckow S., Szafrański S.P., Grischke J., Qu T., Doll-Nikutta K., Steglich M., Yang I., Häussler S., Stiesch M. (2024). Structure and Composition of Early Biofilms Formed on Dental Implants Are Complex, Diverse, Subject-Specific and Dynamic. npj Biofilms Microbiomes.

[7] Quirynen M., Bollen C.M., Eyssen H., van Steenberghe D. (1994). Microbial Penetration along the Implant Components of the Brånemark System. An in Vitro Study. Clin. Oral Implant. Res..

[8] Son M., Song Y., Yu Y., Kim S.Y., Huh J.-B., Bae E.-B., Cho W.-T., Na H.S., Chung J. (2023). The Oral Microbiome of Implant-Abutment Screw Holes Compared with the Peri-Implant Sulcus and Natural Supragingival Plaque in Healthy Individuals. J. Periodontal Implant Sci..

[9] Sharifi R., Ghaffari T., Ghasemi S., Kafil H.S. (2025). Evaluation of Microgap Size and Microbial Microleakage at the Implant Fixture–Abutment Interface in Original and Compatible Abutments. BioMed Res. Int..

[10] Kellesarian S.V., Javed F., Romanos G.E. (2018). Osteomyelitis Arising Around Osseointegrated Dental Implants: A Systematic Review. Implant Dent..

[11] Garrote M.d.S., Barbosa A.A.C., Teixeira M.d.F.B.M.A., Mendonça E.F., Fenelon G., Guedes O.A., Estrela C. (2025). Severe Chronic Suppurative Osteomyelitis Following Dental Implant Placement. Case Rep. Dent..

[12] Renvert S., Persson G.R., Pirih F.Q., Camargo P.M. (2018). Peri-Implant Health, Peri-Implant Mucositis, and Peri-Implantitis: Case Definitions and Diagnostic Considerations. J. Periodontol..

[13] Lindhe J., Meyle J., Group D of the European Workshop on Periodontology (2008). Peri-Implant Diseases: Consensus Report of the Sixth European Workshop on Periodontology. J. Clin. Periodontol..

[14] Roccuzzo M., Mirra D., Roccuzzo A. (2024). Surgical Treatment of Peri-Implantitis. Br. Dent. J..

[15] Rokaya D., Srimaneepong V., Wisitrasameewon W., Humagain M., Thunyakitpisal P. (2020). Peri-Implantitis Update: Risk Indicators, Diagnosis, and Treatment. Eur. J. Dent..

[16] Bahrami R., Nikparto N., Gharibpour F., Pourhajibagher M., Bahador A. (2024). Antimicrobial Photodynamic Therapy for Managing the Peri-Implant Mucositis and Peri-Implantitis: A Systematic Review of Randomized Clinical Trials. Photodiagn. Photodyn. Ther..

[17] Sculean A., Deppe H., Miron R., Schwarz F., Romanos G., Cosgarea R. (2021). Effectiveness of Photodynamic Therapy in the Treatment of Periodontal and Peri-Implant Diseases. Monogr. Oral Sci..

[18] Baran T.M., Bass D.A., Christensen L., Longbine E., Favella M.D., Foster T.H., Sharma A.K. (2024). Safety and Feasibility of Photodynamic Therapy for Percutaneous Image-Guided Abdominopelvic Abscess Drainage: Phase 1 Trial. Radiology.

[19] Jerjes W., Upile T., Hamdoon Z., Alexander Mosse C., Morcos M., Hopper C. (2011). Photodynamic Therapy Outcome for T1/T2 N0 Oral Squamous Cell Carcinoma. Lasers Surg. Med..

[20] Shen X., Dong L., He X., Zhao C., Zhang W., Li X., Lu Y. (2020). Treatment of Infected Wounds with Methylene Blue Photodynamic Therapy: An Effective and Safe Treatment Method. Photodiagn. Photodyn. Ther..

[21] Wirth K.T., Wang E.Y., Hawryluk D.S., Byrne M.M., Arca M.J., Wakeman D.S., Pegoli W., Darcy D.G., Pavelka M.S., Wilson N.A. (2025). Photodynamic Therapy with Methylene Blue Effectively Kills Antibiotic Resistant Bacteria from Pediatric Patients with Perforated Appendicitis. Sci. Rep..

[22] Zhang W., Chen S., Bai Z., Gan M., Chen M., Zhang Y., Liu S., Liu D. (2024). Photodynamic Therapy for Oral Squamous Cell Carcinoma: Current Status, Challenges, and Prospects. Int. J. Nanomed..

[23] Tardivo J.P., Del Giglio A., de Oliveira C.S., Gabrielli D.S., Junqueira H.C., Tada D.B., Severino D., de Fátima Turchiello R., Baptista M.S. (2005). Methylene Blue in Photodynamic Therapy: From Basic Mechanisms to Clinical Applications. Photodiagn. Photodyn. Ther..

[24] Correia J.H., Rodrigues J.A., Pimenta S., Dong T., Yang Z. (2021). Photodynamic Therapy Review: Principles, Photosensitizers, Applications, and Future Directions. Pharmaceutics.

[25] Cwalinski T., Polom W., Marano L., Roviello G., D’Angelo A., Cwalina N., Matuszewski M., Roviello F., Jaskiewicz J., Polom K. (2020). Methylene Blue—Current Knowledge, Fluorescent Properties, and Its Future Use. J. Clin. Med..

[26] Barnes T.G., Hompes R., Birks J., Mortensen N.J., Jones O., Lindsey I., Guy R., George B., Cunningham C., Yeung T.M. (2018). Methylene Blue Fluorescence of the Ureter during Colorectal Surgery. Surg. Endosc..

[27] Mahmoudi H., Bahador A., Pourhajibagher M., Alikhani M.Y. (2018). Antimicrobial Photodynamic Therapy: An Effective Alternative Approach to Control Bacterial Infections. J. Lasers Med. Sci..

[28] Vaishampayan A., Grohmann E. (2021). Antimicrobials Functioning through ROS-Mediated Mechanisms: Current Insights. Microorganisms.

[29] Hong Y., Zeng J., Wang X., Drlica K., Zhao X. (2019). Post-Stress Bacterial Cell Death Mediated by Reactive Oxygen Species. Proc. Natl. Acad. Sci. USA.

[30] Kligman S., Ren Z., Chung C.-H., Perillo M.A., Chang Y.-C., Koo H., Zheng Z., Li C. (2021). The Impact of Dental Implant Surface Modifications on Osseointegration and Biofilm Formation. J. Clin. Med..

[31] Lehmann K., Kadler G., Kalyanov A., Schweizer T.A., Walt H., Essig H. (2025). Zirconium Dental Implants as Potential Optical Waveguides in Photodynamic Inactivation of Bacterial Biofilms—A Pilot Study. Microorganisms.

[32] Liebermann A., Freitas Rafael C., Colle Kauling A.E., Edelhoff D., Ueda K., Seiffert A., Maziero Volpato C.A., Güth J.-F. (2018). Transmittance of Visible and Blue Light through Zirconia. Dent. Mater. J..

[33] Maharishi A., McLaren E.A., White S.N. (2024). Color- and Strength-Graded Zirconia: Strength, Light Transmission, and Composition. J. Prosthet. Dent..

[34] Qaryan M., Kafian-Attari I., Afara I.O. (2025). Monte Carlo Simulations of Light-Skin Interactions: Implications for Therapeutic and Oncological Applications. Photodiagn. Photodyn. Ther..

[35] Dedes G., Parodi K. (2016). Monte Carlo Simulations of Particle Interactions with Tissue in Carbon Ion Therapy. Int. J. Part. Ther..

[36] Harrison R.L. (2010). Introduction To Monte Carlo Simulation. AIP Conf. Proc..

[37] Lee H. (2024). Monte Carlo Methods for Medical Imaging Research. Biomed. Eng. Lett..

[38] Meister D., Boksansky J., Guthe M., Bittner J. (2020). On Ray Reordering Techniques for Faster GPU Ray Tracing. Proceedings of the Symposium on Interactive 3D Graphics and Games.

[39] Finlayson L., McMillan L., Suveges S., Steele D., Eftimie R., Trucu D., Brown C.T.A., Eadie E., Hossain-Ibrahim K., Wood K. (2024). Simulating Photodynamic Therapy for the Treatment of Glioblastoma Using Monte Carlo Radiative Transport. J. Biomed. Opt..

[40] Valentine R.M., Wood K., Brown C.T.A., Ibbotson S.H., Moseley H. (2012). Monte Carlo Simulations for Optimal Light Delivery in Photodynamic Therapy of Non-Melanoma Skin Cancer. Phys. Med. Biol..

[41] Guo D., Wang A., Xie T., Zhang S., Cao D., Sun J. (2020). Effects of Ex Vivo Ischemia Time and Delayed Processing on Quality of Specimens in Tissue Biobank. Mol. Med. Rep..

[42] Sanchez W.Y., Prow T.W., Sanchez W.H., Grice J., Roberts M.S. (2010). Analysis of the Metabolic Deterioration of Ex Vivo Skin from Ischemic Necrosis through the Imaging of Intracellular NAD(P)H by Multiphoton Tomography and Fluorescence Lifetime Imaging Microscopy. J. Biomed. Opt..

[43] Lasers for Medical Applications. https://www.sciencedirect.com/book/edited-volume/9780857092373/lasers-for-medical-applications.

[44] Jacques S.L. (2013). Optical Properties of Biological Tissues: A Review. Phys. Med. Biol..

[45] Nelder J.A., Mead R. (1956). A Simplex Method for Function Minimization. Comput. J..

[46] Ghaiyoomi S., Shokouhi Mostafavi S.K., Manavi A., Lawaf S., Azizi A. (2025). Investigating the Effect of Photodynamic Therapy With a 660 Nm Laser With Methylene Blue and Cold Atmospheric Plasma Therapy on Streptococcus Sanguinis. J. Lasers Med. Sci..

[47] Youf R., Müller M., Balasini A., Thétiot F., Müller M., Hascoët A., Jonas U., Schönherr H., Lemercier G., Montier T. (2021). Antimicrobial Photodynamic Therapy: Latest Developments with a Focus on Combinatory Strategies. Pharmaceutics.

[48] Wainwright M., Maisch T., Nonell S., Plaetzer K., Almeida A., Tegos G.P., Hamblin M.R. (2017). Photoantimicrobials-Are We Afraid of the Light?. Lancet Infect. Dis..

[49] Azizi B., Budimir A., Bago I., Mehmeti B., Jakovljević S., Kelmendi J., Stanko A.P., Gabrić D. (2018). Antimicrobial Efficacy of Photodynamic Therapy and Light-Activated Disinfection on Contaminated Zirconia Implants: An in Vitro Study. Photodiagn. Photodyn. Ther..

[50] Bergmann F., Foschum F., Marzel L., Kienle A. (2021). Ex Vivo Determination of Broadband Absorption and Effective Scattering Coefficients of Porcine Tissue. Photonics.

[51] Firbank M., Hiraoka M., Essenpreis M., Delpy D.T. (1993). Measurement of the Optical Properties of the Skull in the Wavelength Range 650–950 nm. Phys. Med. Biol..

[52] Kothuri S.K., MacMahon D., Lanka P., O’Flynn C., Henn P., Andersson-Engels S., Gautam R., Konugolu Venkata Sekar S. (2025). Broadband Time Domain Diffuse Optical Characterization of Human Cadaver Bone from 500 to 1100 nm. Sci. Rep..

[53] Pifferi A., Torricelli A., Taroni P., Bassi A., Chikoidze E., Giambattistelli E., Cubeddu R. (2004). Optical Biopsy of Bone Tissue: A Step toward the Diagnosis of Bone Pathologies. J. Biomed. Opt..

[54] Konugolu Venkata Sekar S., Pagliazzi M., Negredo E., Martelli F., Farina A., Dalla Mora A., Lindner C., Farzam P., Pérez-Álvarez N., Puig J. (2016). In Vivo, Non-Invasive Characterization of Human Bone by Hybrid Broadband (600–1200 nm) Diffuse Optical and Correlation Spectroscopies. PLoS ONE.

[55] Bashkatov A.N., Genina E.A., Kochubey V.I., Tuchin V.V. (2005). Optical Properties of Human Skin, Subcutaneous and Mucous Tissues in the Wavelength Range from 400 to 2000 nm. J. Phys. D Appl. Phys..

[56] Boujnah M., Ennaceri H., Kenz A., Benyoussef A., Chavira E., Loulidi M., Ez-Zahraouy H. (2020). The Impact of Point Defects on the Optical and Electrical Properties of Cubic ZrO_2_. J. Comput. Electron..

[57] Oftadeh R., Perez-Viloria M., Villa-Camacho J.C., Vaziri A., Nazarian A. (2015). Biomechanics and Mechanobiology of Trabecular Bone: A Review. J. Biomech. Eng..

[58] Hasenleitner M., Plaetzer K. (2019). In the Right Light: Photodynamic Inactivation of Microorganisms Using a LED-Based Illumination Device Tailored for the Antimicrobial Application. Antibiotics.

[59] Soares J.M., Yakovlev V.V., Blanco K.C., Bagnato V.S. (2024). Photodynamic Inactivation and Its Effects on the Heterogeneity of Bacterial Resistance. Sci. Rep..

[60] Astuti S.D., Hafidiana, Rulaningtyas R., Abdurachman, Putra A.P., Samian, Arifianto D. (2020). The Efficacy of Photodynamic Inactivation with Laser Diode on Staphylococcus Aureus Biofilm with Various Ages of Biofilm. Infect. Dis. Rep..

[61] Araújo N.C., Fontana C.R., Bagnato V.S., Gerbi M.E.M. (2014). Photodynamic Antimicrobial Therapy of Curcumin in Biofilms and Carious Dentine. Lasers Med. Sci..

[62] Sprawls P. (1995). Physical Principles of Medical Imaging.

[63] Bashkatov A.N., Genina E.A., Tuchin V.V. (2011). Optical Properties of Skin, Subcutaneous, and Muscle Tissues: A Review. J. Innov. Opt. Health Sci..

[64] Fretwurst T., Müller J., Larsson L., Bronsert P., Hazard D., Castilho R.M., Kohal R., Nelson K., Iglhaut G. (2021). Immunohistological Composition of Peri-Implantitis Affected Tissue around Ceramic Implants—A Pilot Study. J. Periodontol..

[65] Silva E., Félix S., Rodriguez-Archilla A., Oliveira P., Martins dos Santos J. (2014). Revisiting Peri-Implant Soft Tissue—Histopathological Study of the Peri-Implant Soft Tissue. Int. J. Clin. Exp. Pathol..

[66] Ghorbani J., Rahban D., Aghamiri S., Teymouri A., Bahador A. (2018). Photosensitizers in Antibacterial Photodynamic Therapy: An Overview. Laser Ther..

