# Supplementary material for: From Simulation to Therapy: Light Guidance Through Zirconium Dental Implants for Bone-Targeted Photodynamic Inactivation
Source: Bioengineering (Basel). 2026 Jul 30;13(8):880. doi: 10.3390/bioengineering13080880 (PMC13510031; doi:10.3390/bioengineering13080880)
Supplement: Supplementary file 1 [file bioengineering-13-00880-s001.zip › bioengineering-4413381-supplementary.pdf]

Supplementary Material of " From Simulation to Therapy: Light Guidance Through Zirconium Dental Implants for Bone-Targeted Photodynamic Inactivation"

Table S1. Underlying Data from MCRT simulations for Approximation curves.

**Human**	Mean Radius R (mm) for a defined Power per Area Threshold (mW/cm^2)															
**PC**	**90**	**80**	**70**	**60**	**50**	**40**	**35**	**30**	**25**	**20**	**18**	**16**	**14**	**12**	**10**	**9**	**8**
1	4.4	4.4	4.5	5.0	5.6	5.4	5.8	6.1	6.9	7.3	7.8	8.2	8.6	8.5	9.0	9.2	9.6
2	6.7	5.2	5.0	5.0	4.7	4.3	4.8	5.5	5.9	6.9	7.5	7.8	8.4	9.0	10.1	10.4	10.1
3	3.2	3.3	3.3	3.5	3.9	4.2	4.0	4.6	5.0	6.3	6.8	7.5	7.8	7.8	8.3	8.8	9.2
**PC**	**7**	**6**	**5**	**4**	**3**	**2**	**1**	**0.9**	**0.8**	**0.7**	**0.6**	**0.5**	**0.4**	**0.3**	**0.2**	**0.1**	
1	9.9	10.2	10.6	11.1	12.0	12.4	13.5	13.5	13.8	14.3	15.0	15.4	16.1	16.7	17.4	23.2	
2	10.4	10.6	11.8	13.0	13.0	13.9	14.1	14.0	14.2	14.4	15.0	16.1	16.6	17.1	18.0	22.2	
3	9.5	9.7	9.7	10.0	9.9	10.6	12.5	12.8	13.2	13.4	13.8	14.3	15.2	16.3	17.1	18.9	

**porcine**	Mean Radius R (mm) for a defined Power per Area Threshold (mW/cm^2)															
**PC**	**90**	**80**	**70**	**60**	**50**	**40**	**35**	**30**	**25**	**20**	**18**	**16**	**14**	**12**	**10**	**9**	**8**
1	4.6	4.7	4.7	4.9	5.8	5.9	6.4	6.4	7.3	8.0	8.3	8.5	8.9	9.1	9.3	9.6	10.0
2	5.2	6.0	5.0	5.0	5.3	4.8	4.9	5.5	6.5	7.4	7.9	8.6	9.0	9.4	10.2	10.5	10.9
3	3.4	3.8	4.0	4.3	4.4	4.9	5.1	5.8	6.9	7.9	7.9	8.1	8.5	8.8	9.0	9.1	9.4
**PC**	**7**	**6**	**5**	**4**	**3**	**2**	**1**	**0.9**	**0.8**	**0.7**	**0.6**	**0.5**	**0.4**	**0.3**	**0.2**	**0.1**	
1	10.4	11.0	11.1	11.9	12.6	13.2	15.5	15.7	15.9	16.7	17.0	17.6	17.9	19.0	21.1	27.5	
2	11.1	11.9	12.6	12.8	13.4	14.1	16.2	16.5	16.6	16.8	17.5	18.2	18.4	19.4	21.3	26.5	
3	9.6	9.8	9.8	10.7	11.5	12.7	15.0	15.4	15.9	16.4	16.9	17.4	18.1	18.8	20.3	22.2	

Table S2. Exponential Fitting Equations for Approximation curves determined from **Table S1**.

Exponential Fitting Equation for Approximation Curves		
**PC**	**human**	**porcine**
1	y = 425.21 * EXP(-0.421 * x)	y = 279.16 * EXP(-0.345 * x)
2	y = 408.73 * EXP(-0.405 * x)	y = 280.59 * EXP(-0.338 * x)
3	y = 293.02 * EXP(-0.432 * x)	y = 259.64 * EXP(-0.362 * x)
